# Effect of Soy Milk Consumption on Quality of Life in Iranian Postmenopausal Women 

**Published:** 2015-06

**Authors:** Mansoreh Nourozi, Fedyeh Haghollahi, Fatemeh Ramezanzadeh, Parichehr Hanachi

**Affiliations:** 1 Valie-Asr Reproductive Health Research Center, Tehran University of Medical Science, Tehran, Iran; 2 Department of Biology, Biochemistry Unite, Faculty of Basic Sciences, Alzahra University, Tehran, Iran

**Keywords:** Quality of Life, Post menopausal, Soy Milk, MENQOL

## Abstract

**Objective:** To find out whether or not soy milk as a phytoestrogen product can improve the quality of life of the Iranian postmenopausal women.

**Materials and methods:** Participants of this randomized clinical trial were 57 healthy postmenopausal women. All eligible women were randomly divided into two groups of soy milk (SG) and control (CG). Individuals in the SG (n = 34) received 500 ml soy milk including genistein (28.86 mg/dl) and daidzein (8.25 mg/dl) per day, while the participants in the CG (n = 23) received 500 ml low fat cow milk per day during 8 months. Both groups also took daily calcium-D capsules (500 mg calcium and 200 IU D3). The quality of life of all participants was examined twice (at the baseline and the end of the eighth month) using the menopause-specific quality of life (MENQOL) questionnaire.

**Results:** A total of 57 healthy postmenopausal women with a mean age of 52.13 (3.05) years were included in this study. Despite the significant but weak difference was observed between SG and CG in the sexual domain score (the mean of percent change: 0.46% vs. 33.94%, respectively; p = 0.031), while significant relationship was found between the soy milk consumption and improvement in the domains studied (vasomotor, psychosocial and physical).

**Conclusion:** Overall our findings showed that soy milk does not improve the quality of life in postmenopausal women. But to achieve more reliable results, it is recommended further study to be done with a larger sample size, more prolonged, and with participants having severer vasomotor symptoms.

## Introduction

The most recent definition of quality of life presented by World Health Organization (WHO) (QOL) is “the individual’s perception of their position in life in the context of culture and value systems in which they live and in relation to their goals, expectations, standards and concerns" (1).

 Although menopause is not a disease, it is a natural event happening in middle-aged women. However, the cessation of ovarian activity and subsequent hormonal changes (especially in the absence of estrogen) lead to several problems that can affect the quality of life in postmenopausal women (2-4).

The problems experienced by postmenopausal women are of varying degrees (depending on the years after menopause, hormonal interventions, ethnicity, lifestyle, etc.) that generally include vasomotor instability (hot flashes, flushing, and sweating), sleep disturbance, depression, atrophic changes (atrophy in vaginal and vulvar epithelium, generalized atrophy of the skin, and urinary tract problems), osteoporosis and cardiovascular disease, and partly change in sexual desire (5).

Several studies have found that a sufficient dose of estrogen (hormone replacement therapy) is effective in mitigating the symptoms of menopause and improving the quality of life (6-9). However, in a study by Rossouw et al., 2002, they have cast doubt on the protective effects of hormone therapy in reducing the risk of coronary heart disease in postmenopausal women and confirmed the increased risk of stroke, invasive breast cancer, deep vein thrombosis and pulmonary embolism in consumers of hormone replacement (10). Therefore, researchers were encouraged to look for an alternative therapy that would not only be risk-free, but would also compensate for the estrogen deficiency.

Given the obvious differences in the incidence of hot flashes between postmenopausal women in Asia and their Western counterparts (10 to 25 percent of Chinese and Indonesian women and 58 to 93 percent of Western women) (11), it appears that these differences are at least partly associated with a diet rich in plant estrogens (phytoestrogens) in Asian countries (11).

Phytoestrogens are found naturally in many plants. They stick to estrogen receptors and perform different estrogenic and non-estrogenic activities (5). Soybeans are a rich source of phytoestrogens like isoflavones that includes genistein, daidzein and glycitein. Daily consumption in Asian countries is much higher than Western nations (1 to 36 g in Japan, Korea, Taiwan and Indonesia vs. 4 mg in United States) (11). In addition, there is evidence suggesting that the positive effect of soy products on vasomotor symptoms (12-14), maturation of vaginal epithelium (15), bone formation and resorption markers (14, 16, 17), lipoproteins (14, 18), visual memory (19), nonverbal short-term memory, and frontal lobe function (20) at the age of menopause. However, there are studies negating the findings above. Hence the consumption of soy and its products as an alternative treatment to hormone therapy is discussed here. 

Despite findings that suggest soy products have no impact on menopausal women's quality of life (21-23), there is some evidence indicating an improvement in the quality of life of menopausal women who consume soybeans (11) or its protein extracted (24). The study shows that the consumption of cow milk enriched with 50 mg of soy isoflavones can reduce vasomotor symptoms and improve quality of life among postmenopausal women (16).

Soy milk is a derivative of soybeans. Research into its effects on postmenopausal complications has been limited. Soy milk as a stable emulsion of oil, water and soy protein is made by soaking dried beans and grinding them with water. One of the advantages of these nutrients compared to other soybean products is that in addition to isoflavones, it has a composition similar to that of cow milk that can meet part of the individual’s needed minerals such as calcium and iron (25).

This study aims to find out whether or not soy milk can act as a simple, affordable solution to middle-aged Iranian menopausal women by providing them with phytoestrogens and probably diminishing menopausal symptoms in them, and in turn improving the quality of their life.

## Materials and methods

This study was performed as a randomized clinical trial among 264 postmenopausal women aged between 45 and 60 in the Vali-e-asr Reproductive Research Center, the Endocrine and Metabolism Research Center and the Women Research Centerat Alzahra University, Tehran, Iran, between 2009 and 2011. Before commencing the study, the survey was approved by the Ethics Committee of Tehran University of Medical Science and volunteers were informed verbally and in writing of the objectives of the study and how it would be conducted. 

**Figure 1:**
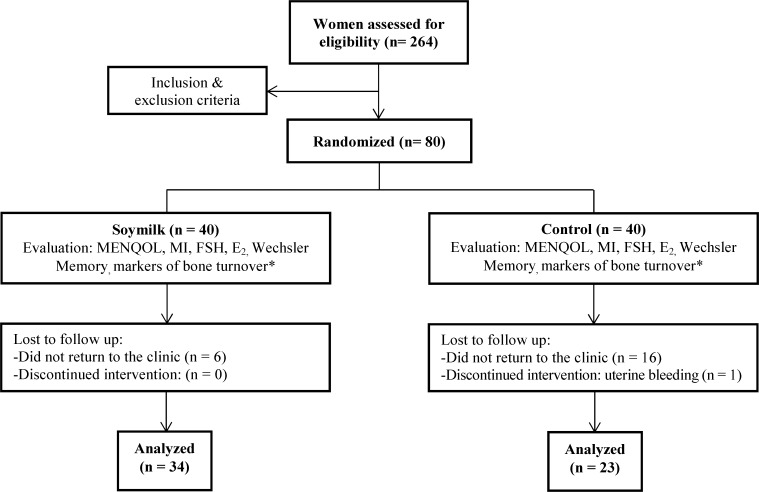
Study flow chart illustrates enrollment, number of women in the intent-to-treat population, randomization to treatment groups, and follow-up of study participants

Of the 264 healthy postmenopausal asymptomatic women living in Tehran, Iran, who volunteered to participate in the study, eighty were selected. The written informed consent was obtained from all (Figure 1).

All the women underwent medical examination (interviews, hematology, biochemistry, gynecologic examination, Papanicolaou test, mammography, and transvaginal ultrasonography) in order to determine patient eligibility. 

Inclusion criteria included a lapse of 1 to 7 years since the last menstruation and at least the daily occurrence of two vasomotor symptoms (hot Flashes and sweats). Those with FSH < 40 and Lack of menstruation < 12 months were excluded from the study. They were also asked if they had had treatment with hormone replacement therapy (HRT) (during the last 6 months), cholesterol-lowering, anti osteoporotic, or other interfering drugs (i.e. tibolone), if they had used any soy products (during the last 2 years), if they had diabetes or history of cancer, or if they were on a vegetarian diet. They were also examined for menopausal symptoms requiring therapy, and drug or nicotine addiction. The participants were also asked to say if they had a routine exercise program.

In brief, after the 80 recruited women completed the Beck Depression Inventory (BDI), they were randomized into following two groups: the Intervention group [40 women: 500 milliliter soy milk (MAXOY co, Iran) daily], and the Control Group (40 women: 500 milliliter low fat cow milk daily). Both groups also received daily calcium-D capsules (500 mg calcium and 200 IUD3, Pharma Drau., Iran). The patients had knowledge of the treatment assignment. Groups were obliged to record the daily number of hot flashes (the first week of study) and consumption of milk and capsules (during the8-month study) in a form.

The quality of life of the participants was examined on two occasions (at the baseline and the end of the eighth month). The interviewer used the standardized menopause-specific quality of life (MENQOL) questionnaire (26). The reliability and validity of this questionnaire has been approved previously by Iranian researchers in the translated version (27, 28).

MENQOL is a 29-item questionnaire including four domains that evaluates the quality of life in postmenopausal women. The Domains include vasomotor (3 items), psychosocial (7 items), physical (16 items), and sexual (3 items). Each domain is scored separately. There is no overall score that can be obtained from this questionnaire since the relative contribution of each domain to an overall score is unknown.

*MENQOL = Menopause Specific Quality of Life; MI = maturation index; FSH = Follicle Stimulating Hormone; E_2 _= Estradiol; Markers of bone turnover = Osteocalcin &CTX-beta

For all items, there is eight-point scale, ranging from one (no experience of problem) to eight (the most severe problem), suggesting higher scores indicate worse quality of life (26). The questionnaires were presented by a skilled person and the participants were requested to answer the questions carefully.

Throughout the study, Maxsoy Co. agreed to supply all the soy milk needed (the simple type). The type and quantity of isoflavones was measured by means of the HPLC system at the biomedical laboratory at Al-Zahra University’s Center for Research on Women’s Affairs. The concentrations of genistein and daidzein were determined as 28.86 mg per deciliter (± 66.0) and 8.25 milligrams per deciliter (± 13.1), respectively (29). In other words, each person consumed 500 ml of soy milk, about 185.55 mg of isoflavones (144.3 mg genistein and 41.25 mg daidzein) on a daily basis.

***Statistical analysis***

The data analysis was performed using version 17 of the SPSS (SPSS; SPSS Inc., Chicago, IL, USA) software. The mean and standard deviation (SD) scores were calculated for numerical variables, and the number and percentage for categorical variables. The p value < 0.05 was considered statistically significant. A confidence level of 95% and test power of 80% was considered. To examine whether the two groups were matched, the Chi-square test and nonparametric Mann-Whitney were used. In addition, a comparison was done of the two groups in terms of the effects of soy milk using the Mann-Whitney nonparametric test, and the change percent of domain score was then computed after intervention using the following formula: % change = [(post test score-pretest score) ÷ pretest score] × 100.In each group, the Wilcoxon nonparametric test was used to compare the pretest and post-test.

## Results

In this study, of 264 volunteer postmenopausal women, 80 were eligible, of which 34 in the soy milk group (SG) and 23 in the control group (CG) completed the study (Figure 1) and 23 participants were excluded for various reasons. The demographic data is shown in table 1. The two groups were matched for age, weight, height, body mass index (BMI), age at first menstruation (menarche), the duration of menopause, number of pregnancies, serum follicle-stimulating hormone (FSH) level, the test scores of depression (BDI) and the frequency of hot flashes per week In the SG, 50% were employed and64.7% had a high school diploma or higher education, while in the CG, 34.2% were employed and 47.8% had a high school diploma or higher degrees. But between the two groups in terms of occupation and education, there was no significant difference (p = 0.256 and p = 0.163, respectively, not reported in the table).

According to table 2, in both groups, after the intervention, the mean score of vasomotor domain decreased significantly (p = 0.011 in SG, p = 0.011 in CG). But the mean percentage change (Table 3), after comparing the two groups, were not significant statistically (-22.82 vs. -9.86 respectively, p = 0.501). The mean score of sexual domain no significantly decreased in SG (12.04 to 9.35, p = 0.058) and significant increased in the CG (6.85 to 9.54, p = 0.018). In contrast to the CG, the non-significant reduction was observed in the mean score of psychosocial and physical domains in SG. As mentioned previously, decreased score represents an improvement in quality of life and increased score indicates deterioration. Therefore an improvement was observed in the vasomotor domain in both groups and deterioration in the sexual domain in CG.

**Table 1:** Comparison of demographic characteristics between the two groups

Variables	Soy Milk group Mean (SD)	Control group Mean (SD)	p-value*
**Age (year)**	**52.13 (3.05)**	**51.39 (2.89)**	**0.165**
**Weight (Kg)**	**68.55 (9.31)**	**68.07 (10.55)**	**0.827**
**Height (Cm)**	**154.69 (5.76)**	**154.68 (5.94)**	**0.987**
**BMI**	**28.68 (3.84)**	**28.64 (4.14)**	**0.873**
**Menarche (year)**	**13.5 (1.67)**	**13.64 (1.81)**	**0.909**
**Duration of menopause (year)**	**2.74 (1.6)**	**2.8 (1.72)**	**0.862**
**Gravida**	**3.47 (2.18)**	**4.0 (1.76)**	**0.556**
**FSH (mIu/ml)**	**81.97 (23.78)**	**81.67 (23.52)**	**0.981**
**BDI**	**10.13 (9.52)**	**14.21 (9.62)**	**0.135**
**Hot flashes (frequency per week)**	**26.06 (29.34)**	**24.6 (30.95)**	**0.664**

* Mann-Whitney Test

**Table 2:** Comparison the pretest and post-test scores in each domain for two groups separately (Mean and Standard deviation)

Domains of quality of life	Group					
	Soy milk			Control		
	Before intervention	After intervention	p-value*	Before intervention	After intervention	p-value*
**Vasomotor ** **Mean (SD)**	**13.36 (6.20)**	**10.43 (6.34)**	**0.011** §	**12.50(6.05)**	**9.29 (5.47)**	**0.010§**
**Psychosocial ** **Mean (SD)**	**21.68 (12.53**	**18.52 (10.03)**	**0.398**	**20.58 (8.05)**	**22.33 (5.23**	**0.432**
**Physical** **Mean (SD)**	**54.67 (27.74)**	**53.83 (21.04)**	**0.831**	**45.73 (12.43)**	**52.27 (11.0)**	**0.172**
**Sexual** **Mean (SD)**	**12.04 (7.80)**	**9.35 (5.73)**	**0.058**	**6.85 (3.58)**	**9.54 (6.64)**	**0.018** §

* Wilcoxon Test;

§ Significant < 0.05

**Table 3:** Comparison of the percentage change in each domain score between the two groups after intervention

Domains of quality of life	Mean of % change		p-value*
	Soy milk	Control	
**Vasomotor **	**-9.86**	**-22.82**	**0.501**
**Psychosocial **	**-1.15**	**27.68**	**0.296**
**Physical **	**7.77**	**22.03**	**0.550**
**Sexual**	**0.46**	**33.94**	**0.031** §

* Mann-Whitney Test;

§ Significant < 0.05

The mean percentage change of the vasomotor domain score (Table 3), after comparing the two groups, were not significant statistically (-9.86 in SG, -22.82 in CG, p = 0.501). Also in the CG compared with SG, the mean percentage changes in the other domains scores (psychosocial, physical and sexual) increased as well as the mean scores. However, comparing the two groups, the difference of the mean percentage change in the sexual domain score is only significant (0.46 in SG, 33.94 in CG, p = 0.031).

## Discussion

In early menopause, vasomotor symptoms such as hot flashes and night sweats; somatic symptoms such as fatigue, physical pain, and vaginal dryness; and psychological symptoms such as irritability, anxiety, depression, decreased libido and difficulty sleeping are often reported. The number and duration of vasomotor symptoms vary among different populations. The average length of flashing is six months to five years; however, in 20% of the cases, they may continue until the age 70 or 80 (30).Williams et al. have done a study on2,703 American postmenopausal women between the ages of 40 and 65. Results showed that hot Flashes affects work (46.0%), social activities (44.4%), leisure activities (47.6%), sleep (82.0%), mood (68.6%), concentration (69.0%), sexual activity (40.9%), and total energy level (63.3%),hence decreases the overall quality of life by as much as 69.3% (31).

The probability of this risk in women with severe hot flashes is 3.58 against women with low to moderate hot flashes. Therefore, effective and safe treatment that reduces the symptoms can improve the quality of life (31).

Studies show that vasomotor symptoms are clearly associated with lower estrogen levels. Thus estrogen therapy in postmenopausal women significantly improves hot flashes and sweating. The results of studies on the effects of soy products on menopausal symptoms and the quality of life in postmenopausal women are very different. The reason for this difference is mostly because of the non-uniform conditions such as differences in age range of the subjects and duration of the menopausal age, the number and severity of hot flashes, sample size, duration of intervention, and different products of soy isoflavones consumed. In addition, the studies conducted to assess menopausal symptoms have generally observed a significant placebo effect in reducing hot flashes (5, 11, 13).

Also, the metabolism of isoflavones depends on intestinal flora and the dietary intake of antibiotics. Yet there are individual differences. Only a third of people have the ability to produce equol (the most potent metabolite of soy derived from daidzein) (32). The amount of isoflavones in soy food products differs, depending on the type of soybeans, harvest year and geographic locations (33). Therefore, such studies are faced with limitations and their results should be compared while keeping the afore-mentioned restrictions in mind.

Apart from this study, there have been at least seven other studies that evaluated the quality of life in postmenopausal women who consume soy milk (11, 20-24, 34). In the three studies, this association was significant, but non-significant in other four studies. It appears that differences in products used to intervene are the main factor leading to the differences in results. However, in some cases, even with the use of the same product, different results can be observed.

In the study conducted by García-Martín, milk enriched with isoflavones extracted from soy was used (23), whereas in the studies done by Amato (20) and Ho (21), only isoflavones extracted were utilized. However, in the García-Martín’s study, the quality of life improved, and in the other two, it didn’t make a difference. Soy protein was studied with different values in the studies of Basaria (24), Welty (11) and Kok (23). In the studies by Basaria (24) and Welty (11), soy protein was successful, whereas in the study by Kok (23), it failed to improve the quality of life. Also, soy flour was used in the Lewis’s study, but no difference was found between groups in terms of hot flashes and the quality of life (34).

In present study, differences in the quality of life in postmenopausal women consuming soy milk and the control group were not significant. However, in each group, significant improvement in vasomotor domain was reported. Still there was no difference between them, which does not indicate little or no effect of soy milk on the vasomotor domain. But it seems that it may indicate a need to increase the duration of the study to establish the effects of soy milk and to have proper selection of samples, in terms of the number and severity of hot flashes. There is evidence indicating a significant reduction in hot flashes in the control group (even to the extent of 50%) (5) And greater effectiveness of soy and its products in women with more severe vasomotor symptoms (11).

However, this study showed significant but weak effect of soy milk on the sexual domain as well as non-significant improvement in the Psychosocial and physical aspects were found. While in the control group, in the rest of the domains except the vasomotor, there was a non-significant reduction in the quality of life. It is recommended that further study to be done with a larger sample size, more prolonged, and with participants who have severer vasomotor symptoms. 
